# Oral Manifestations in Geriatric Patients: An Observational Study in Western Maharashtra

**DOI:** 10.7759/cureus.71977

**Published:** 2024-10-20

**Authors:** Prerna M Jadhav, Ashwinirani S Renukaradhya

**Affiliations:** 1 Department of Oral Medicine and Radiology, School of Dental Sciences, Krishna Vishwa Vidyapeeth (Deemed to be University), Karad, IND

**Keywords:** diabetes, geriatric, hypertension, periodontitis, systemic diseases

## Abstract

Objective

The current study aims to assess various oral changes and associated systemic diseases in geriatric patients.

Methodology

A prospective, observational study was conducted among geriatric patients visiting the Department of Oral Medicine and Radiology School of Dental Sciences, Krishna Vishwa Vidyapeeth (KVV), Karad. A total of 100 patients aged 60 and above were screened. The study was conducted over a six-month period, from January 2024 to June 2024. Data recorded in a clinical proforma included essential demographic information such as age and gender, personal habits like smoking and alcohol, and medical history. All the patients were clinically examined under standard conditions, utilizing a mouth mirror, probe, explorer, and retractors under proper illumination. Hard tissue findings, including caries, fractures, restorations, and periodontal conditions, were meticulously documented, while soft tissue findings included evaluations of the gums and oral mucosa for lesions. The oral mucosal examination was done using the World Health Organization (WHO) guide. Histopathologic examination was done in relevant cases to confirm the final diagnosis. Data entry was done using SPSS (17.0 version) Java (TM) Platform SE binary (IBM Corp, London, UK) for Windows. To check the association between data, the chi-square test was applied.

Results

Out of 100 elderly patients, 57 were males and 43 were females. Hypertension was identified as the most prevalent condition, affecting 32% of patients. Tobacco consumption was most commonly reported among males, affecting 49% of the patients. Prevalent hard tissue oral manifestations were periodontitis (29.5%) followed by partial edentulism (27%), dental caries (25.5%), wasting diseases (15.5%), and complete edentulism (2.5%), while soft tissue findings were gingivitis (46.7%) followed by oral cancer (28.5%), leukoplakia (7.1%), candidiasis (7.1%), lichen planus (7.1%), and irritational fibroma (3.5%).

Conclusion

The most prevalent hard tissue finding was periodontitis, while the most common soft tissue finding was gingivitis, followed by oral cancer. Older adults are particularly vulnerable to tobacco use, vitamin deficiencies, and stress, emphasizing the need for appropriate care. It is crucial to implement programs aimed at enhancing both general and oral health in the geriatric population.

## Introduction

Old age is regarded as an inevitable biological phenomenon. Oral health is as important as general health, but in old age, it is definitely difficult to maintain oral health. Geriatric dentistry aims to provide dental services to the elderly. Geriatric patients are vulnerable to long-term diseases and multiple symptoms due to a decline in body functions; hence, dentists should be aware of various oral manifestations in geriatrics. According to the World Health Organization (WHO), a person aged 60 years and above is considered geriatric. In India, people aged 60 years and above in 2022 (as of July 1) comprise around 10.5% of the country’s population [1].

The frequency of oral cancer in India is high due to variations in cultural, ethnic, and geographic factors, as well as lifestyle habits [2]. Elderly individuals often have systemic diseases, making them more susceptible to conditions affecting both the hard and soft tissues of the oral cavity [3]. Changing lifestyles, particularly shifts in dietary habits and increased sedentary behavior, have led to a rise in diabetes, hypertension, and other systemic diseases. In light of this, the present study aims to assess the oral manifestations in geriatric patients and explore their association with systemic diseases, ultimately enhancing the overall health of this population.

## Materials and methods

This cross-sectional, hospital-based observational study comprised geriatric patients visiting the Outpatient Department (OPD) of the Department of Oral Medicine and Radiology at the School of Dental Sciences, Krishna Vishwa Vidyapeeth (KVV), Karad.

Ethical approval

Ethical approval for this study was obtained from the Institutional Ethical Committee (IEC) of Krishna Vishwa Vidyapeeth (KVV), Karad (Protocol Number: 033/2024-2025). The research adhered to the ethical standards set forth by the Declaration of Helsinki [4].

Sample size calculation

The sample size (n) was determined using the following formula:

n = z² p q/L²

where p is the proportion of hard tissue findings = 74%, q = 100 - P = 26%, L is the allowable error = 10%.

Substituting the values: n = (1. 96)² × 74 × 26/100, n ≈ 74.

A total of 74 patients was calculated as the sample size.

Selection criteria

Inclusion criteria: (1) Patients aged 60 years and older visiting the Department of Oral Medicine and Radiology. (2) Patients who consented to participate in the study. Exclusion criteria: (1) Patients who declined to participate in the study. (2) Patients with mental health conditions.

A total of 100 geriatric patients aged 60 years and older were enrolled in the study after providing written consent. Participants were recruited through consecutive sampling as they visited the outpatient department and were divided into three age groups: 60-69 years (group 1), 70-79 years (group 2), and 80 years and older (group 3). The study was conducted from January 2024 to June 2024.

Data were collected using a clinical proforma that included demographic information, personal habits like smoking and alcohol use, and medical histories, which included self-reported systemic diseases verified through previous medical records.

The clinical examination of all the patients was done under standard conditions, utilizing a mouth mirror, probe, explorer, and retractors under proper illumination and findings related to hard tissues (caries, fractures, and restorations) and soft tissues (lesions and inflammation) were meticulously documented. The oral examination assessed probing depth, clinical attachment levels, and gingival inflammation to evaluate periodontitis.

The oral mucosal examination was done using the WHO guide for examination [5]. The lesions were diagnosed based on standard recommendations and criteria [6,7]. The International Classification of Diseases: Application to Dentistry and Stomatology (ICD‑DA) was used for the collection and reporting of common oral diseases and conditions [8]. For suspicious lesions, biopsy samples were collected under local anesthesia, ensuring complete excision for analysis. Histopathological evaluations utilized hematoxylin and eosin (H&E) staining and additional special stains, such as Papanicolaou, when necessary for definitive diagnosis. Cancer staging adhered to the criteria set by the American Joint Committee on Cancer (AJCC).

Data entry was done using SPSS (17.0 version) Java (TM) Platform SE binary (IBM Corp., London, UK) for Windows. To check the association between data, the chi-square test was applied.

## Results

Out of 100 elderly patients, 57 were males and 43 were females. These patients were distributed into three groups based on their age groups. Group 1 included patients of age group of 60-69 years. Group 2 included patients of age group 70-79 years. Group 3 included patients of age group >80 years.

**Figure 1:**
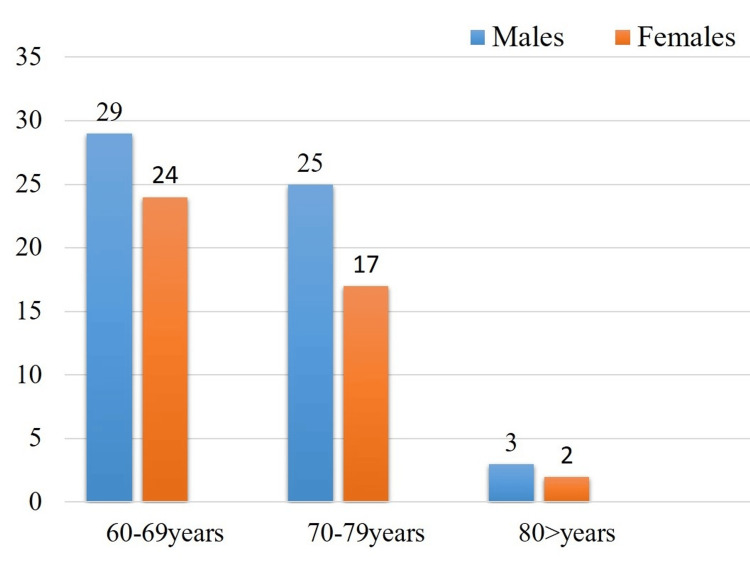
Gender and age distribution among 100 geriatric patients.

Figure 1 illustrates the distribution of gender and age among 100 geriatric patients. In group I, which includes patients aged 60-69 years, there were 29% males and 24% females. Group II, comprising those aged 70-79 years, included 25% males and 17% females. In group III, which consisted of patients aged 80 years and above, there were 3% males and 2% females. The data indicate that the majority of the population (53%) aware of their dental and medical conditions fell within the 60-69 age range.

Table 1 presents the distribution of various systemic diseases among the 100 geriatric patients in the study. Hypertension was identified as the most prevalent condition, affecting 32% of patients. This was followed by cardiovascular diseases, which were present in 14% of the patients, and diabetes, impacting 9%. Additionally, a combined prevalence of diabetes and hypertension was noted in 4% of the individuals.

**Table 1:** Distribution of systemic diseases among 100 geriatric patients. Group 1 included patients of age group of 60-69 years. Group 2 included patients of age group 70-79 years. Group 3 included patients of age group >80 years.

Medical conditions	Group 1	Group 2	Group 3	Total
Hypertension	22	10	0	32
Cardiovascular diseases	9	4	1	14
Diabetes	4	4	1	9
Combined diabetes and hypertension	2	1	1	4
Total	37	19	3	59

**Figure 2:**
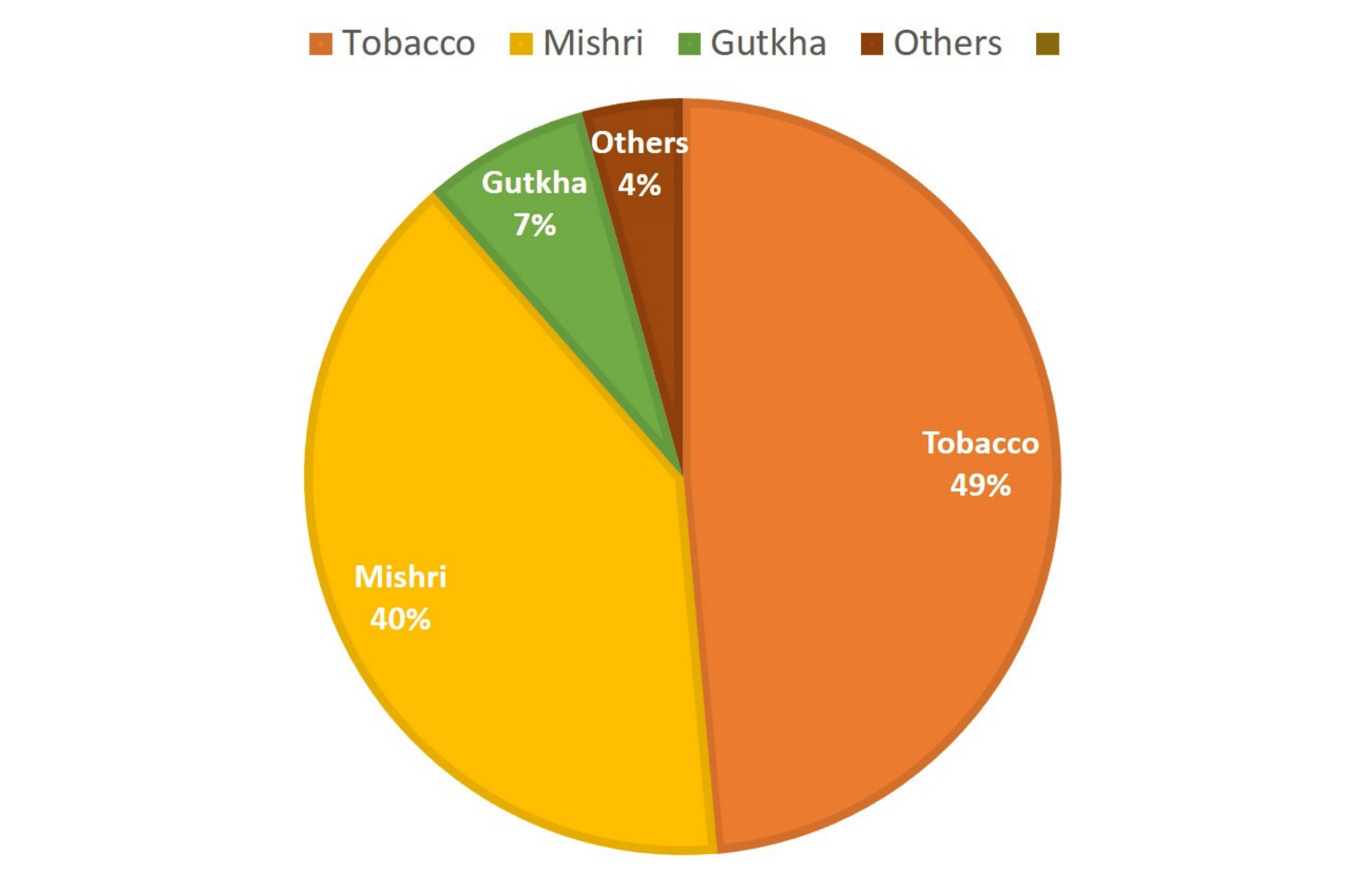
Distribution of habits among 100 geriatric patients.

Figure 2 describes the patterns of substance use among the participants in the present study. Tobacco consumption was most commonly reported among males, affecting 49% of the patients. In contrast, Mishri consumption accounted for approximately 40% and was predominantly observed in females. The use of gutkha was noted in 7% of the population, while 4% of patients reported other habits, including alcohol, cigarettes, and bidis.

Table 2 outlines the oral manifestations observed in the study, categorized into hard and soft tissue findings. Among the hard tissue findings, periodontitis was the most prevalent condition, affecting 72% of the patients, followed by partial edentulism at 66%. Dental caries was present in 62% of the patients, while wasting diseases affected 38%. Complete edentulism was noted in 6% of the individuals, highlighting the significant oral health challenges faced by geriatric patients.

**Table 2:** Analysis of hard tissue findings in 100 geriatric patients. Group 1 included patients of age group 60-69 years. Group 2 included patients of age group 70-79 years. Group 3 included patients of age group >80 years.

S. no	Clinical oral manifestations	Group 1	Group 2	Group 3	Total
1.	Periodontitis	49	20	3	72
2.	Partial edentulism	44	19	3	66
3.	Dental caries	47	14	1	62
4.	Wasting diseases	23	14	1	38
5.	Complete edentulism	3	2	1	6

**Figure 3:**
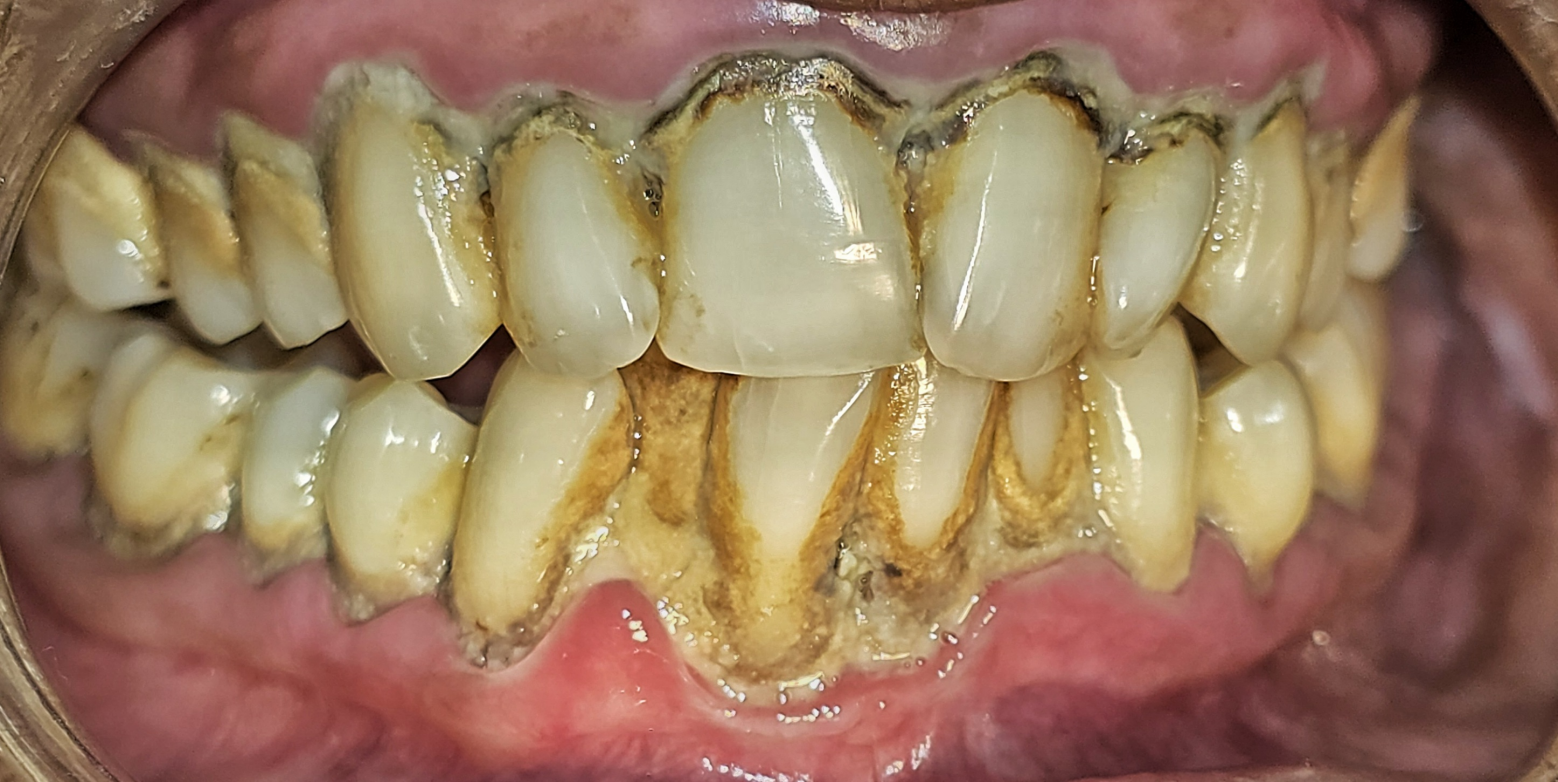
Figure shows periodontitis in a 62-year-old male patient.

Among hard tissue findings, the most prevalent condition was periodontitis. Figure 3 shows periodontitis in a 62-year-old male patient.

**Figure 4:**
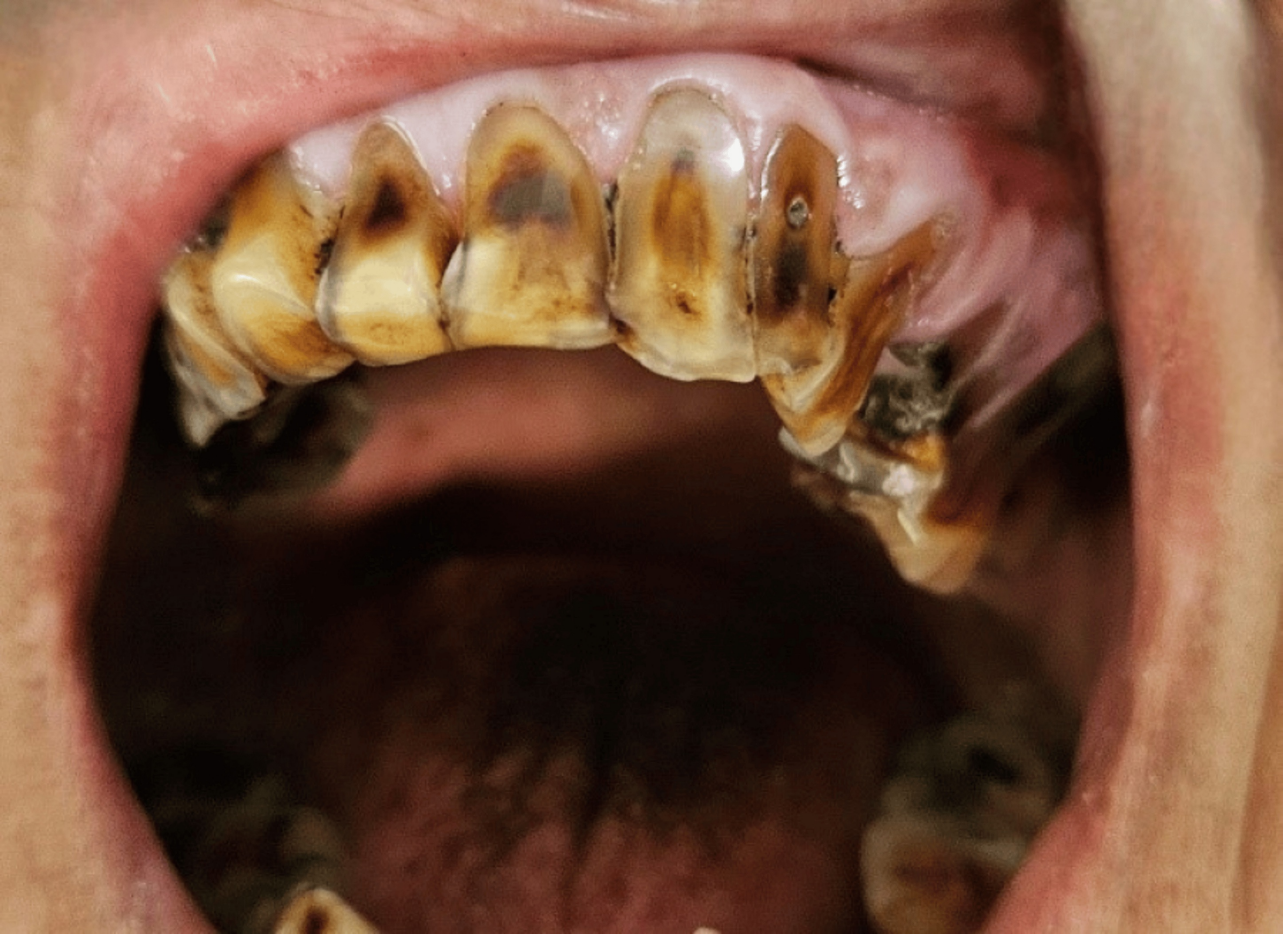
Figure shows wasting disease in a 67-year-old female patient.

Figure 4 shows wasting disease (generalized attrition) in a 67-year-old female patient.

**Figure 5:**
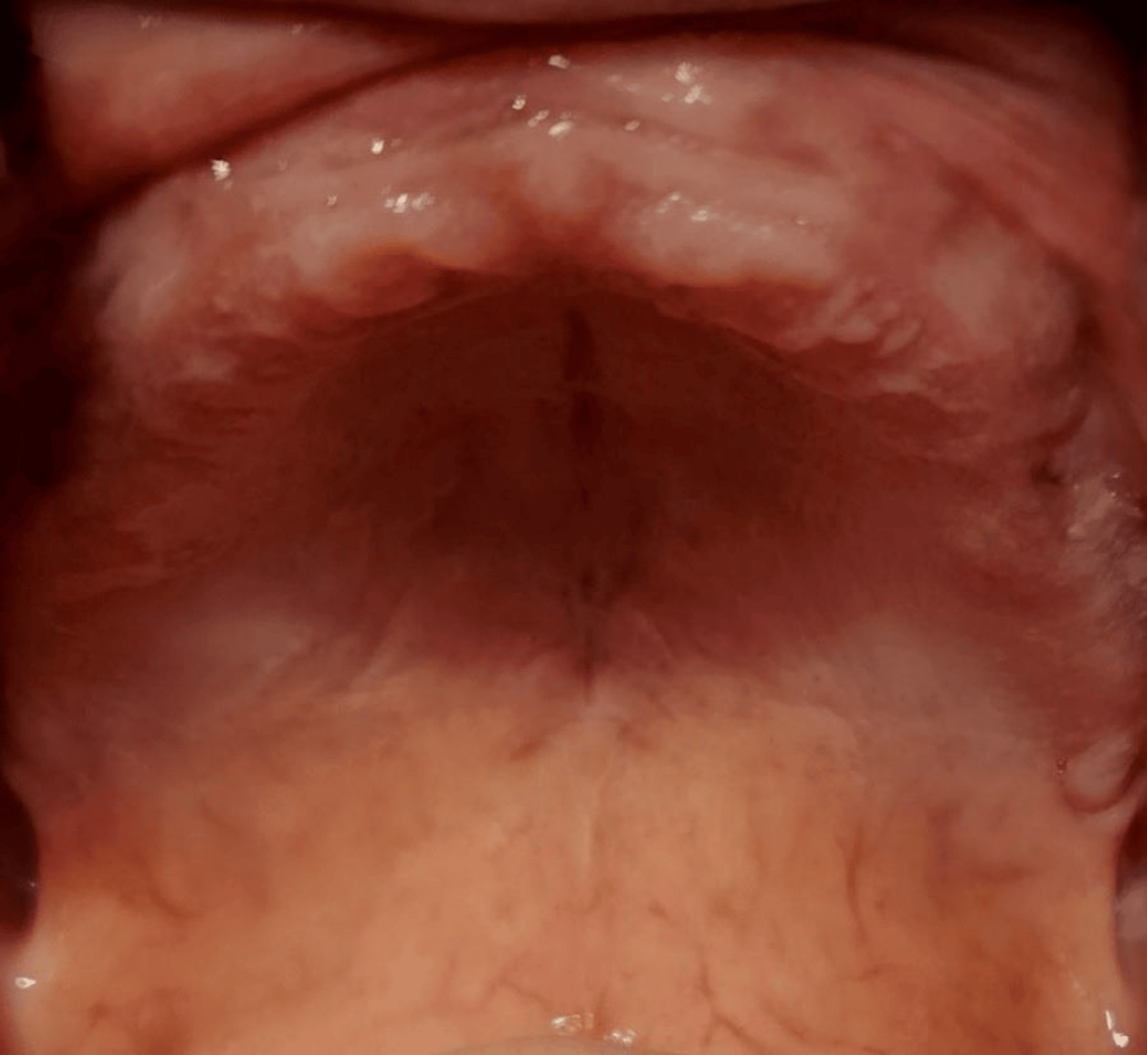
Figure shows a 63-year-old male patient with complete edentulism in the maxillary arch.

Figure 5 illustrates a 63-year-old male patient presenting with complete edentulism in the maxillary arch. This condition, characterized by the complete loss of teeth in the upper jaw, highlights the challenges faced in oral health among geriatric patients.

Table 3 presents the prevalent soft tissue findings observed in the study. Gingivitis was the most common condition, affecting 13% of the participants, followed by oral cancer, which was noted in 8%. Leukoplakia, candidiasis, and lichen planus were each identified in 2% of the patients, while irritational fibroma was recorded in 1%. This data underscores the significant soft tissue challenges encountered by geriatric patients.

**Table 3:** Analysis of soft tissue findings in 100 geriatric patients. Group 1 included patients of age group of 60-69 years. Group 2 included patients of age group 70-79 years. Group 3 included patients of age group >80 years.

S. no	Clinical oral manifestations	Group 1	Group 2	Group 3	Total
1.	Gingivitis	12	1	0	13
2.	Oral cancer	6	2	0	8
3.	Leukoplakia	1	1	0	2
4.	Candidiasis	1	1	0	2
5.	Lichen planus	0	2	0	2
6.	Irritational fibroma	1	0	0	1

**Figure 6:**
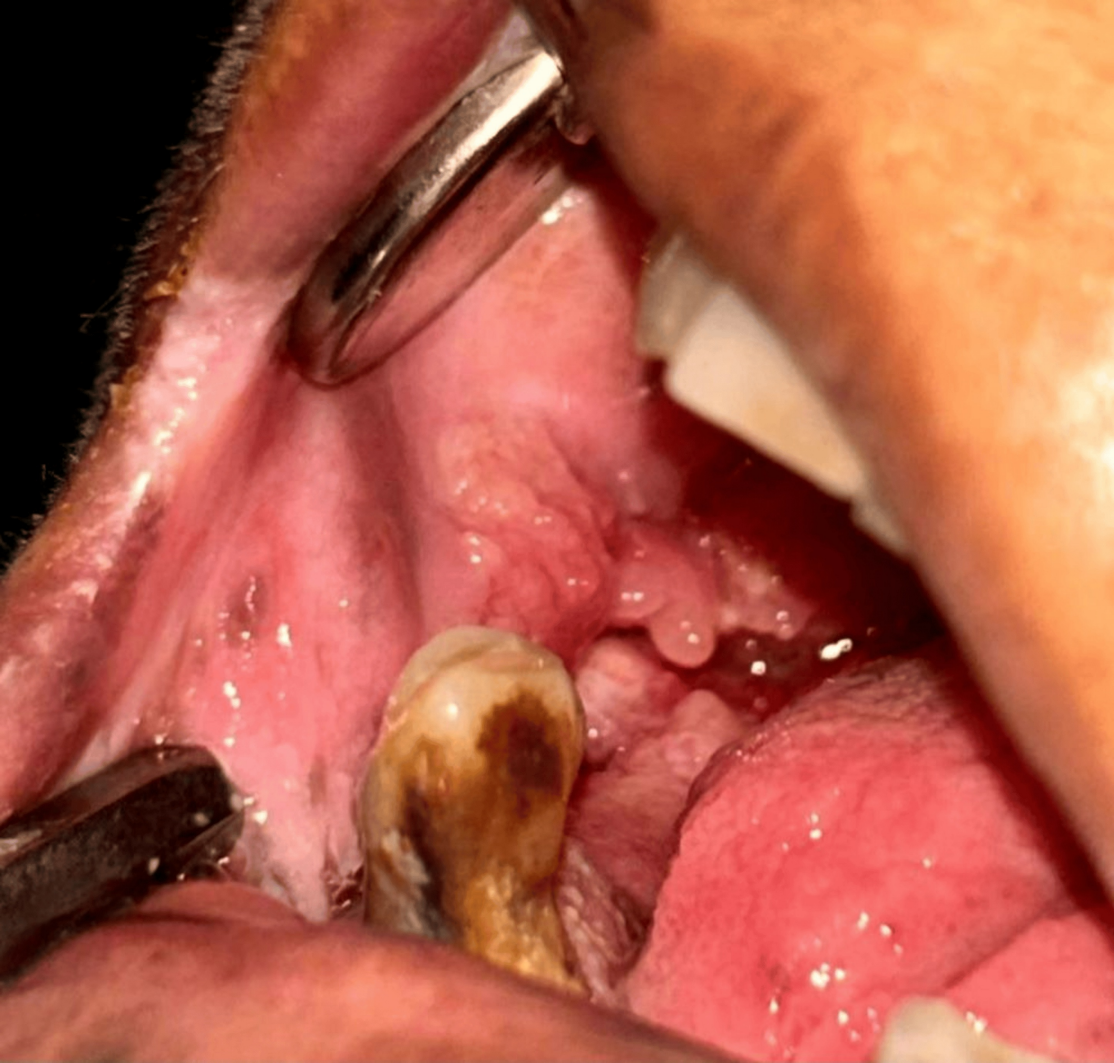
Figure shows a 65-year-old male patient with a malignancy involving the right buccal mucosa and vestibule.

Figure 6 illustrates a 65-year-old male patient with a malignancy affecting the right buccal mucosa and vestibule. This image highlights the clinical presentation of oral cancer, emphasizing the importance of early diagnosis and treatment.

**Figure 7:**
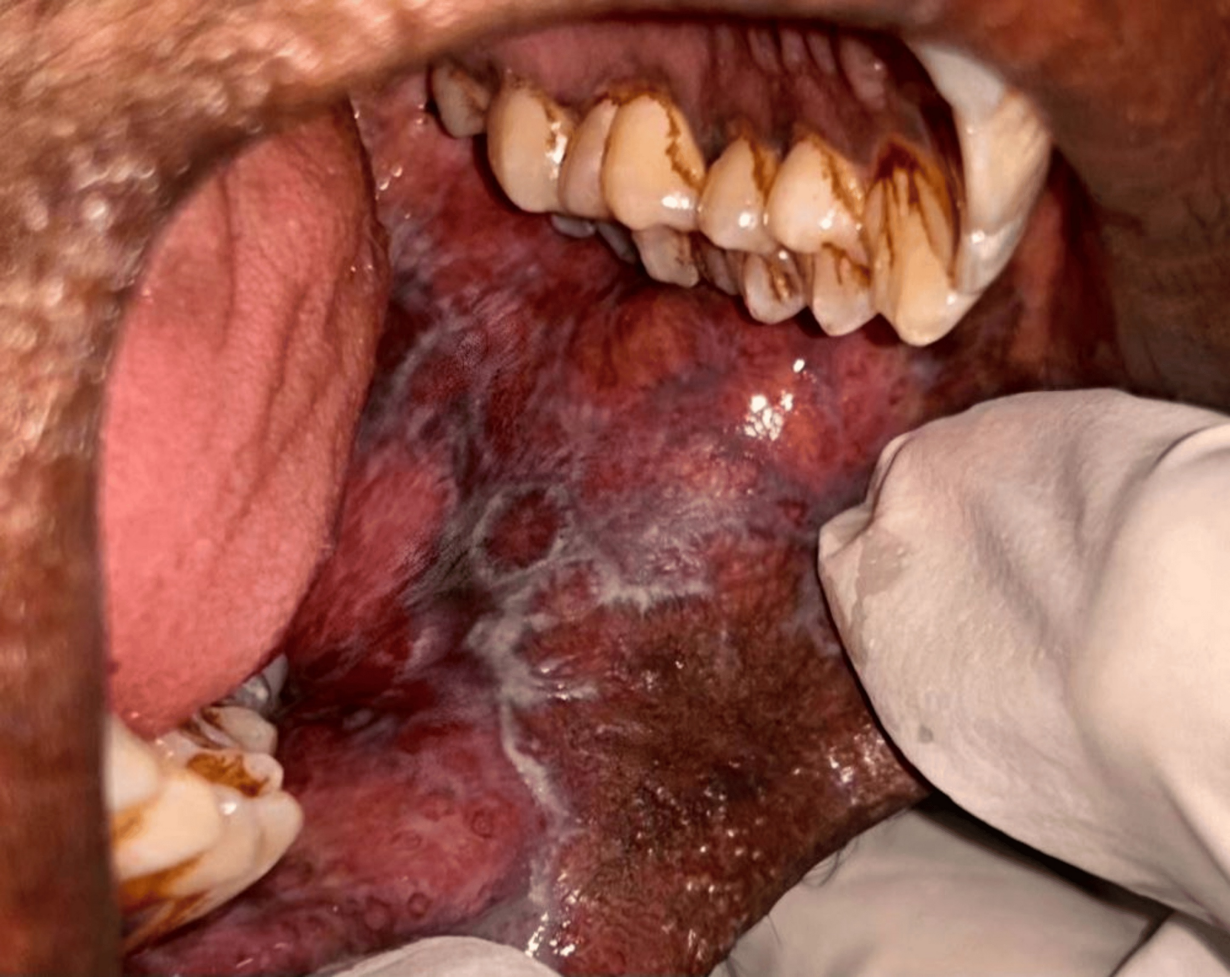
Figure shows a 75-year-old male patient with lichen planus on the left buccal mucosa.

Figure 7 shows a 75-year-old male patient with lichen planus on the left buccal mucosa. This condition is often associated with chronic inflammation and can impact the patient's oral health.

## Discussion

The oral cavity experiences significant age-related changes that impact the mucosal, dental, and periodontal tissues, potentially resulting in various oral pathologies. Contributing factors include cognitive decline, disabilities, unhealthy lifestyle choices such as smoking and alcohol consumption, as well as poor oral hygiene, all of which can lead to a decline in oral health among older adults [9].

Recent demographic analyses indicate a worldwide increase in the elderly population, with similar trends observed in India. The World Health Organization (WHO) cites two primary factors driving this trend: rising life expectancy and declining fertility rates [10].

Several epidemiological studies have enhanced our understanding of the prevalence, extent, and severity of oral diseases within the aging population. While much research has focused on oral squamous cell carcinoma, our study examines a broader range of lesions. As the global elderly population increases, it is essential to prioritize the maintenance of both oral and systemic health to enhance quality of life.

In the present study, out of 100 patients, 57% were male and 43% were female, yielding a male-to-female ratio of 1.4:1. This contrasts with findings by Pratik and Desai, who reported a ratio of 70% males to 30% females among 300 subjects [11], and Rohini et al., who found 73.3% males and 26.6% females among 75 participants in Chennai [12].

The present study indicates that the majority of oral manifestations occurred in patients aged 60-69 years. This aligns with results from Cheruvathoor et al., who also reported a high prevalence in this age group [13], as did Pratik and Desai in their findings for the 61-70 age group [11].

Tobacco chewing emerged as the most prevalent habit in 49% of patients. This is consistent with Cheruvathoor et al.'s findings, which indicated a prevalence of 74% for tobacco chewing among their patients [13]. Hypertension was the most common systemic condition identified in our study (32%), consistent with findings from Devi and Manikandan (46.3%) [14] and Kambampati et al. (17.5%) [15]. Other systemic conditions noted included cardiovascular diseases (14%) and diabetes (9%), with Denny et al. reporting diabetes at 12.2% and cardiovascular diseases at 2.66% [16].

Systemic diseases and their treatments can have profound effects on oral health, leading to complications such as dental caries, periapical abscesses, tooth loss, and challenges in using dental prostheses. Specifically, antihypertensive medications can lead to oral manifestations, including xerostomia, lichenoid reactions, burning mouth syndrome, loss of taste sensation, and gingival hyperplasia [16].

Our study categorized oral manifestations into hard and soft tissue findings. Edentulism remains a significant issue for older adults, with 66% of our patients classified as partially edentulous, a finding similar to the 74.6% reported by Denny et al. [16]. Periodontitis affects more than 50% of the elderly population. Among the soft tissue findings in our study, gingivitis was observed in 13%, aligning with Denny et al.'s results [16]. We recorded eight cases of oral cancer, primarily affecting the buccal mucosa, which aligns with observations from Naveed [17].

The intensity and duration of tobacco use are directly correlated with the risk of developing oral cancer, making tobacco a significant risk factor [18]. India, being one of the largest consumers of tobacco products, shows high exposure rates among the elderly. The prevalence of leukoplakia is similarly associated with tobacco use, reflecting findings from research by Saraswathi et al. [19,20]. In our study, leukoplakia was predominantly seen in males, corroborating findings by Kumar et al. [21]. Regular oral examinations help in detecting precancerous and cancerous lesions in geriatric patients [22].

Limitations

The limited sample size constrained the scope of our study. As this is a hospital-based investigation, the population attended may not accurately reflect the disease burden in the community. The sample size calculation focused on hard tissue findings, which may limit its adequacy for assessing broader variables such as mucosal changes, systemic diseases, and patient habits. Future studies should include larger samples to better evaluate these factors.

## Conclusions

The present study provides the prevalence and distribution of oral manifestations in the geriatric population of Western Maharashtra. In the present study, the most prevalent hard tissue finding was periodontitis, while the soft tissue finding was gingivitis, and the most commonly found systemic disease was hypertension.

Oral health is a crucial indicator of quality of life in older adults. These individuals often face greater exposure to tobacco products, vitamin deficiencies, and stress, highlighting the need for proper care. It is essential to develop specialized geriatric health services that educate and deliver comprehensive healthcare. Now is the time to implement programs aimed at improving both general and oral health for the geriatric population.
